# AJUBA promotes the proliferation, invasion and migration of NSCLC cells by activating the ERK/β-catenin pathway

**DOI:** 10.1038/s41598-025-98156-z

**Published:** 2025-04-16

**Authors:** Lianyue Qu, Fan Wang, Yuxiang Wang, Zixuan Li

**Affiliations:** 1 Key Laboratory of Diagnostic Imaging and Interventional Radiology of Liaoning Province, Department of Radiology, The First Hospital of China Medical University, Shenyang, P. R. China; 2 Department of Pharmacy, The First Hospital of China Medical University, Shenyang, P. R. China; 3 Department of Radiology, The First Hospital of China Medical University, Shenyang, P. R. China; 4 Department of Interventional Radiology, The First Hospital of China Medical University, Shenyang, P. R. China; 5 Department of Nuclear Medicine, The First Hospital of China Medical University, Shenyang, P. R. China

**Keywords:** AJUBA, NSCLC, β-Catenin, P-ERK, Lung cancer, Cell growth, Cell migration, Cell signalling

## Abstract

Accumulating evidence indicates that AJUBA acts as a potential target for new therapeutics to treat cancers. Nevertheless, the role of AJUBA in non-small cell lung cancer (NSCLC) remains unclear. In the current study, immunohistochemistry (IHC) showed that expression of AJUBA was upregulated in 67.55% of NSCLC tumor samples and was associated with tumor size, lymph node metastasis, advanced tumor stage, poor differentiation and poor prognosis. Loss-of-function assays of AJUBA produced by silencing RNA (siAJUBA) significantly inhibited the proliferation, invasion and migration of H1299 and A549 cell lines. Mechanistically, inhibition of extracellular signal-regulated kinases (ERKs) blocked the AJUBA-induced proliferation, invasion and migration of NSCLC cells, and decreased the expression of proteins related to the endothelial-mesenchymal transition (EMT). Silencing of AJUBA repressed tumor growth and led to a decrease in p-ERK, β-catenin and N-cadherin in vivo. In conclusion,, overexpression of AJUBA facilitates the proliferation and motility of NSCLC cells via the ERK and Wnt/β-catenin pathways. AJUBA may be useful as a prognostic marker which may provide a promising approach for the treatment of NSCLC.

**Supplementary Information:**

The online version contains supplementary material available at 10.1038/s41598-025-98156-z.

## Introduction

According to global cancer statistics, there are more than 2 million newly diagnosed lung cancer patients annually^1^. Despite intensive efforts to treat non-small cell lung cancer (NSCLC) by developing novel therapeutic strategies, lung cancer has the highest mortality rate among all types of cancer globally^2^. Therefore, it will be valuable to explore molecular carcinogenesis and facilitate the development of novel therapeutic targets for NSCLC which can prolong the survival of patients. In mammals, the AJUBA family includes three proteins: AJUBA, Wilms tumor 1 interacting protein, and LIM domain-containing protein 1 (LIMD1). AJUBA, also known as JUB, is located on human chromosome 14 and encodes a protein with a molecular weight of 58 kDa. AJUBA protein is characterized by the presence of three LIM domains at its carboxy terminus, which can assist in its nuclear localization. LIM domains are rich in cysteine and were first discovered in many proteins that regulate cell development. The presence of multiple LIM domains in many proteins implies that AJUBA may bind to them and play a significant role^3^. AJUBA is present in fetal components of the developing placenta and has been shown to be involved in the process of epidermal development^4^. A growing body of research has demonstrated that many proteins which play a key role in development, such as the Fibulin family, 14-3-3β and β-catenin, are also indispensable in a variety of tumors. Therefore, in-depth study of the functions of proteins that play an important role in biological development and their mechanisms in tumors can help develop new strategies for tumor treatment.

Recently, numerous researchers have shown that AJUBA is upregulated in various human tumors^5^. AJUBA promotes colorectal cancer (CRC) growth by inhibiting apoptosis^6,7^.AJUBA has also been shown to stimulate breast cancer cell growth, invasion, chemoresistance and glucose uptake^8,9^. AJUBA is upregulated in human gastric cancers and regulates glucose uptake and mitochondrial function to increase cell growth and chemoresistance^10^. AJUBA is frequently overexpressed in esophageal squamous cell carcinoma tissues and promotes the tumorigenicity, motility and chemoresistance of this cancer^11^. AJUBA is upregulated in hepatocellular carcinoma (HCC), where it promotes proliferation, motility and endothelial-mesenchymal transition (EMT)^12^. AJUBA has been confirmed to be a target gene of certain microRNAs that have tumor-suppressing effects^13^. However, the biological functions of AJUBA in lung cancer have not yet been revealed.

The Wnt/β-catenin pathway is one of the most important molecular pathways in mammals and is overactivated in many tumors, including lung cancer^14^. Previous studies have shown that AJUBA is essential for modulating of the Wnt/β-catenin pathway^15^. Therefore, we hypothesized that AJUBA may facilitate NSCLC progression by the β-catenin pathway.

We analyzed the expression of AJUBA in NSCLC tissues and its clinical significance. The biological roles of AJUBA in NSCLC were also explored. Finally, we found that AJUBA regulates cell proliferation and motility and induces EMT through the ERK and Wnt/β-catenin pathway.

## Results

### AJUBA was upregulated in NSCLC tumor samples and correlated with poor prognosis

**Fig. 1:**
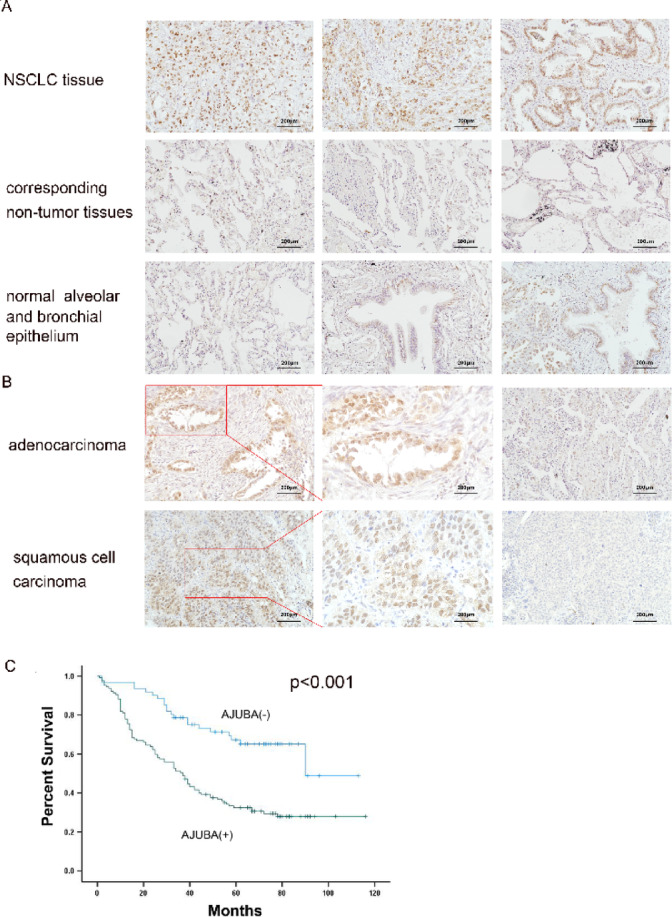
AJUBA expression was upregulated in NSCLC tumor samples and correlated with poor prognosis in NSCLC patients. (**A**) IHC analysis of AJUBA in NSCLC tissue samples and non-tumor tissues (200X). The tissue samples were obtained from three different patients. (**B**) Expression of AJUBA in adenocarcinoma and squamous cell carcinoma (200X). (**C**) Survival rates based on AJUBA were analyzed by the Kaplan-Meier survival method in NSCLC patients.

We investigated AJUBA expression status in 188 sets of cancerous and corresponding paracancerous tissues. AJUBA was expressed at higher levels in tumor tissues (67.55%; 127/188) compared to corresponding normal lung tissues (15.96%; 30/188). AJUBA was negative or weakly positive in most normal alveolar cells and bronchial epithelial cells. Only some bronchi near cancer tissues were AJUBA-positive (Fig. 1, Supplementary Fig. 1). AJUBA was highly expressed in tumor cells in 73.47% (72/98) of adenocarcinomas and in 61.11% (55/90) of squamous cell carcinomas (Fig. 1B). Next, we found no significant link between the expression of AJUBA and factors such as age (*p* = 0.747), gender (*p* = 0.175), and histological type (*p* = 0.710). In contrast, we found that high AJUBA expression was positively associated with tumor size (*p* = 0.004), lymph node metastasis (*p* = 0.011), advanced tumor stage (*p* < 0.001) and poor differentiation (*p* = 0.018; χ2 test, Table 1).

**Table 1:** Correlations between AJUBA expression and clinicopathological factors in lung cancers.

	*N*	AJUBA Negative	AJUBA Positive	*p*-value
Age				
≤ 65	114	38(33.33%)	76(66.67%)	0.747
>65	74	23(31.08%)	51(68.92%)	
Gender				
Male	146	51(34.93%)	95(65.07%)	0.175
Female	42	10(23.81%)	32(76.19%)	
Histological type				
Adenocarcinoma	98	26(26.53%)	72(73.47%)	0.710
Squamous cell carcinoma	90	35(38.89%)	55(61.11%)	
Differentiation				
Well-moderate	116	45(38.79%)	71(61.21%)	**0.018**
Poor	72	16(22.22%)	56(77.78%)	
Tumor stages				
I-II	110	48(43.64%)	62(56.36%)	**<0.001**
III-IV	78	13(16.67%)	65(83.33%)	
T stage				
T1-T2	131	51(38.93%)	80(61.07%)	**0.004**
T3-T4	57	10(17.54%)	47(82.46%)	
Lymph node metastasis				
Negative	95	39(41.05%)	56(58.95%)	**0.011**
Positive	93	22(23.66%)	71(76.34%)	

Subsequently, Kaplan–Meier analysis revealed that among 188 patients, patients with low expression of AJUBA experienced better overall survival (81.445 ± 5.794 months, *p* < 0.001; Fig. 1C), which indicated that AJUBA was correlated with a worse prognosis for NSCLC (51.710 ± 3.893 months). Furthermore, multivariate Cox regression analysis uncovered that advanced TNM stage (*p* = 0.042) and high expression of AJUBA (*p* = 0.002) were independent variables associated with decreased survival rates (Cox proportional risk regression model, Table 2). Collectively, we concluded that AJUBA is overexpressed in NSCLC and is closely related to tumorigenesis and progression.

**Table 2:** Univariate and multivariate analysis of different prognostic features in 188 patients with NSCLC.

	Univariate analysis			Multivariate analysis	
	All cases	HR(95% CI)	*P* value	HR(95% CI)	*P* value
Age			0.085		
≤ 65	114	1.0			
>65	74	1.391			
		(0.956–2.024)			
Gender			0.809		
Male	146	0.948			
		(0.614–1.464)			
Female	42	1.0			
Histological type			0.525		
Adenocarcinoma	98	1.131			
		(0.775–1.649)			
Squamous cell carcinoma	90	1.0			
Differentiation			0.5171		
Well-moderate	116	1.0			
Poor	72	1.136			
		(0.773–1.669)			
Tumor stages			**<0.001**		**0.042**
I-II	110	1.0		1.0	
III-IV	78	2.429		1.635	
		(1.664–3.545)		(1.018–2.627)	
T stage			**0.026**		0.919
T1-T2	131	1.0		1.0	
T3-T4	57	1.564		1.022	
		(1.055–2.320)		(0.667–1.568)	
Lymph node metastasis			**<0.001**		0.131
Negative	95	1.0			
Positive	93	2.067			
		(1.414–3.048)			
AJUBA expression			**<0.001**		**0.002**
Negative	61	1.0		1.0	
Positive	127	2.785		2.206	
		(1.729–4.486)		(1.341–3.629)	

### Downregulation of AJUBA inhibited the growth of NSCLC cells

**Fig. 2:**
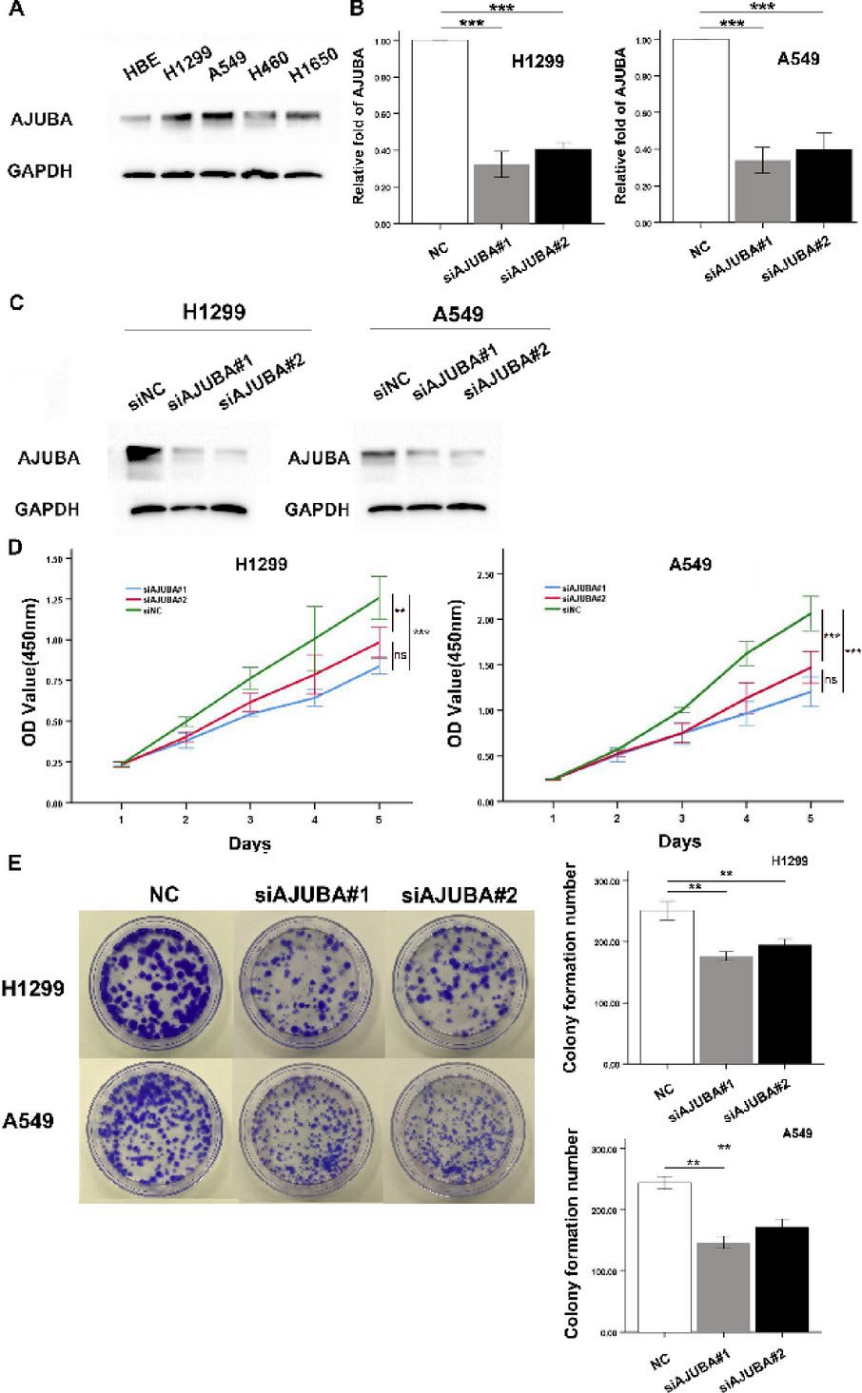
Downregulation of AJUBA attenuated the proliferation of HCC cells. (**A**) Protein expression levels of AJUBA in normal lung epithelial cells (HBE) and NSCLC cell lines (H1299, A549, H460 and H1650). One-way ANOVA was used to analyze the differences in grayscale among different groups. (**B**) The mRNA levels of AJUBA in AJUBA-knockdown H1299 and A549 cells. Two-tailed Student’s t-test was used to analyze the differences between the two groups. NC, negative control. ***P < 0.001. (**C**) The protein levels of AJUBA in AJUBA-knockdown H1299 and A549 cells. One-way ANOVA was used to analyze the differences in grayscale among different groups. (**D**) Cell viability was detected in AJUBA-knockdown H1299 and A549 cells. Effect of AJUBA knockdown on cell growth of H1299 and A549 cells was analyzed by colony formation assay. Two-tailed Student’s t-test was used to analyze the differences between the two groups,. ^**^P < 0 01, ^***^P < 0.001. (**E**) Effect of AJUBA knockdown on cell growth of H1299 and A549 cells was analyzed by colony formation assay. Two-tailed Student’s t-test was used to analyze the differences between the two groups. ^**^P < 0.01.

We next measured the protein levels of AJUBA in four different NSCLC cell lines compared to immortalized human normal HBE cells. Consistent with the in vivo findings, we found the AJUBA was also overexpressed in NSCLC cell lines (Fig. 2A, Supplementary Fig. 2 A). We next silenced AJUBA in H1299 and A549 cells in loss-of-function assays and used Trilencer-27 Universal Scrambled siRNA as a negative control(NC) (Fig. 2B and C, Supplementary Fig. 2B). CCK-8 assays showed that the proliferation of NSCLC cells after siAJUBA treatment decreased in a time-dependent manner (Fig. 2D). The colony-formation capacity of the siAJUBA group was also inhibited compared with the control group (Fig. 2E).

### Downregulation of AJUBA inhibited the invasion and migration of NSCLC cells

**Fig. 3:**
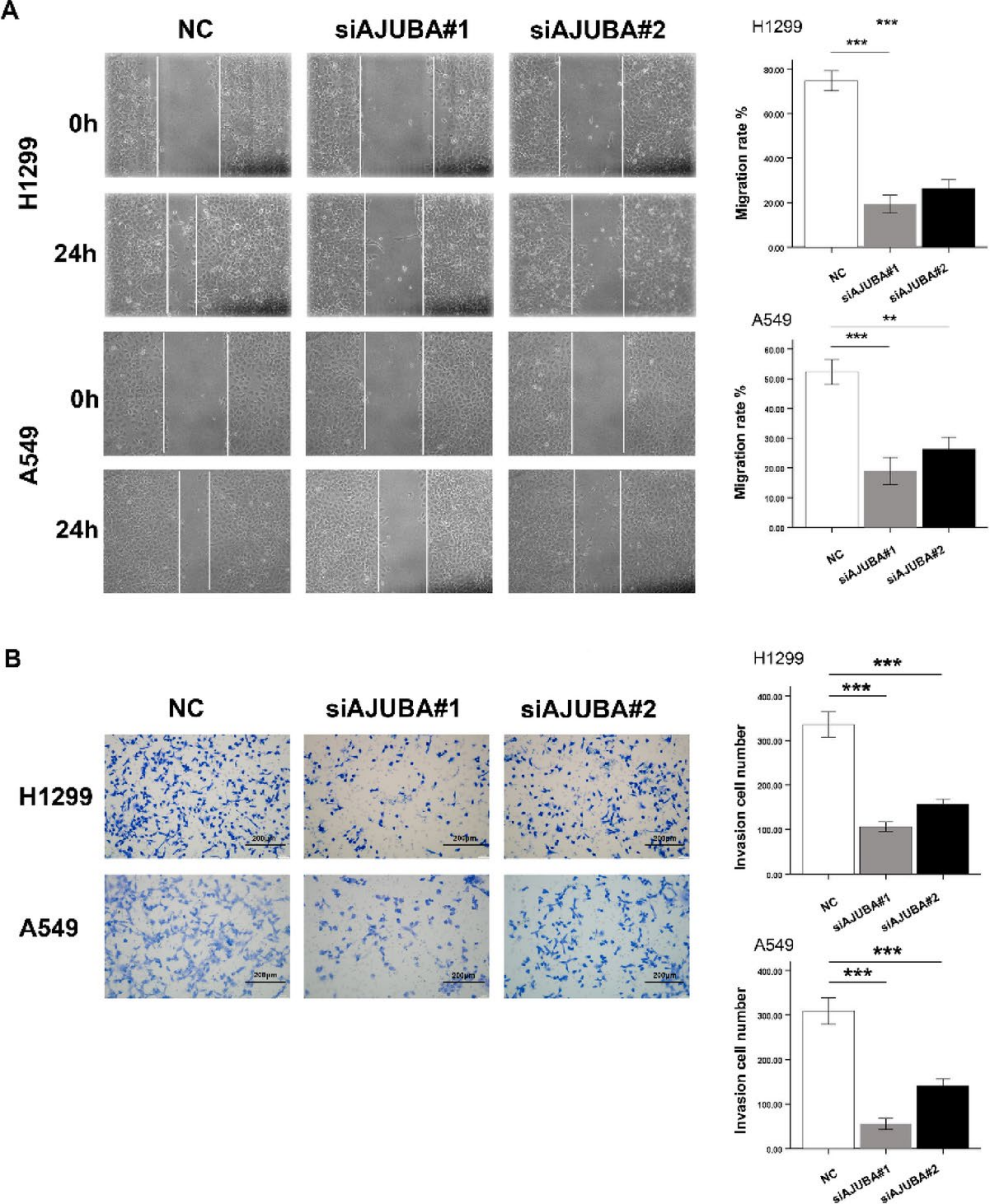
The expression of AJUBA modulated the migration and invasion of NSCLC cells in vitro. (**A**) H1299 and A549 cells were transfected with or without siAJUBA and a wound healing assay was undertaken (100X). Two-tailed Student’s t-test was used to analyze the differences between the two groups. ^**^P < 0.01, ^***^P < 0.001. (**B**) Transwell assay analysis of AJUBA knockdown in H1299 and A549 cells (200X). Two-tailed Student’s t-test was used to analyze the differences between the two groups. ^***^P < 0.001.

To explore the effect of AJUBA on the motility of lung cancer cells, we conducted migration and invasion assays in Transwell plates. After transfection with siAJUBA to downregulate its expression, the migratory capacity of the two cell lines was significantly reduced compared to the control groups (Fig. 3A). In the invasion assay using Matrigel to simulate the in vivo environment, we found that downregulating AJUBA significantly reduced the number of cells penetrating the lower membrane. After statistical analysis, the number of cells that penetrated the membrane in the two siAJUBA groups was significantly lower than in the control groups (Fig. 3B).

### AJUBA was co-expressed with β-catenin in NSCLC tumor samples and its Silencing inhibited the expression of β-catenin and EMT-associated proteins in NSCLC cell lines

**Fig. 4:**
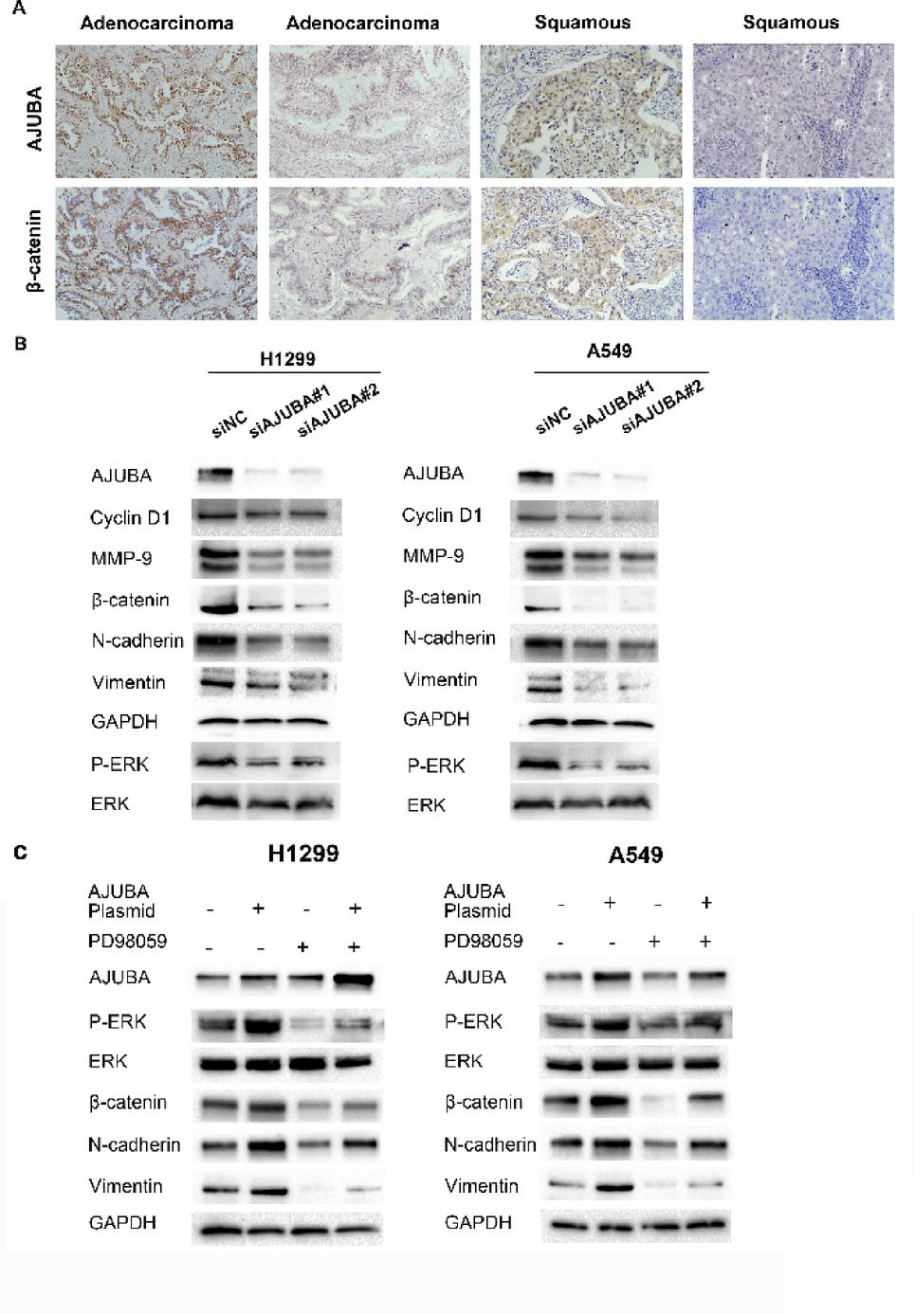
Elevated AJUBA expression was positively associated with β-catenin expression in NSCLC and regulated the expression of EMT-related proteins. (**A**) Correlation of the protein level of AJUBA and β-catenin in NSCLC tumor tissues by IHC (200X). (**B**) Western blotting analysis for AJUBA, Cyclin D1, MMP-9, β-catenin, N-cadherin and Vimentin in H1299 and A549 cells after transfection with or without siAJUBA, GAPDH served as an internal control. One-way ANOVA was used to analyze the differences in grayscale among different groups. (**C**) Western blotting analysis was employed to detect the protein level of AJUBA, p-ERK, β-catenin, N-cadherin and Vimentin in H1299 and A549 cells with AJUBA overexpression and/or PD98059 treatment. One-way ANOVA was used to analyze the differences in grayscale among different groups.

Previous studies have shown that AJUBA can directly bind to β-catenin in hepatocellular carcinoma^16^, and β-catenin also plays a crucial regulatory role in the proliferation and migration of NSCLC. To identify the mechanism of AJUBA-mediated tumor promotion, we used IHC to examine the relationship between AJUBA and β-catenin expression in NSCLC cells of different histology. In Fig. 4A, the expression of AJUBA and β-catenin was positively correlated in both adenocarcinomas and squamous cell carcinomas (χ2 test, Table 3, Supplementary Fig. 3 A). In addition to β-catenin, which mainly acts through the Wnt signaling pathway, other important molecules in this pathway were examined to explore the biological function of AJUBA in NSCLC cell lines. After knockdown of AJUBA, β-catenin, N-cadherin and Vimentin were decreased. Furthermore, Cyclin D1, MMP-9 and p-ERK expression levels were also decreased in both AJUBA-knockdown NSCLC cell lines. These have all been confirmed to be downstream genes in the Wnt pathway. In summary, then, these data indicated that AJUBA positively regulates the Wnt/β-catenin pathway (Fig. 4B, Supplementary Fig. 3B).

**Table 3:** Relationship between the expression of AJUBA and β-catenin in NSCLC.

	All cases	β-catenin negative	β-catenin positive	*p*-value
Adenocarcinoma				**0.012**
AJUBA negative	26	13	13	
AJUBA positive	72	17	55	
Squamous cell carcinoma				**0.014**
AJUBA negative	35	17	18	
AJUBA positive	55	13	42	

### AJUBA upregulated β-catenin via the ERK signaling pathway and regulated EMT progression in NSCLC cells

**Fig. 5:**
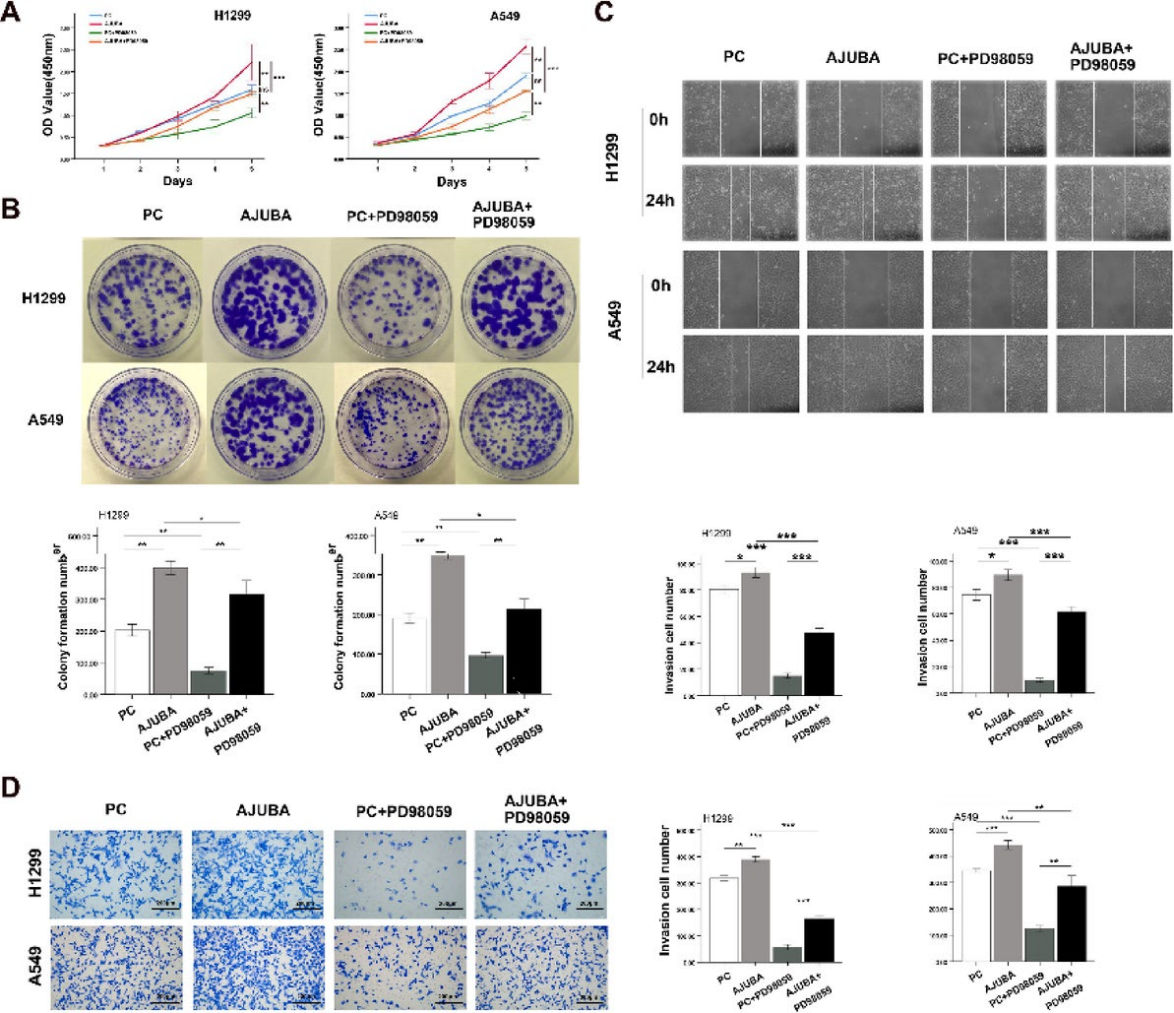
ERK inhibitor PD98059 reversed the proliferation, migration and invasion effects of AJUBA plasmid in lung cancer cells. (**A**) The growth curves of H1299 and A549 cells transfected with AJUBA plasmid and/or PD98059. Two-tailed Student’s t-test was used to analyze the differences between the two groups. ^**^P < 0.01, ^***^P < 0.001. (**B**) The colony formation capacity of H1299 and A549 cells transfected with AJUBA plasmid and/or PD98059. Two-tailed Student’s t-test was used to analyze the differences between the two groups. ^*^P < 0.05; ^**^P < 0.01. (**C**) The migration of H1299 and A549 cells transfected with AJUBA plasmid and/or PD98059 (100X). Two-tailed Student’s t-test was used to analyze the differences between the two groups. ^*^P < 0.05, ^***^P < 0.001. (**D**) The invasion of H1299 and A549 cells transfected with AJUBA plasmid and/or PD98059 (200X). Two-tailed Student’s t-test was used to analyze the differences the between the two groups. ^**^P < 0.01, ^***^P < 0 001.

A previous study showed that the activation of ERK signaling molecules is associated with EMT^17^, ERK1/2 phosphorylates the Ser9 residue of GSK3β, leading to β-catenin upregulation^18^. Therefore, we explored whether AJUBA contributed to EMT via ERK activation. After we upregulated the expression of AJUBA, the expression of β-catenin, N-cadherin and Vimentin were significantly increased; meanwhile the expression level of EMT markers was decreased by the ERK inhibitor PD98059 (Fig. 4C, Supplementary Fig. 3 C). Therefore, ERK inhibition can reverse the AJUBA-induced upregulation of β-catenin and other key proteins in EMT. We next investigated whether AJUBA promotes proliferation and motility via ERK activation. CCK-8, colony formation (Fig. 5A and B), wound healing and cell migration assays (Fig. 5C and D) demonstrated that phosphorylation of ERK inhibitor PD98059 reversed the effects of AJUBA on growth and motility of human lung cancer cells.

### Silencing of AJUBA repressed tumor growth and led to a decrease in p-ERK, β-catenin and N-cadherin in vivo

**Fig. 6:**
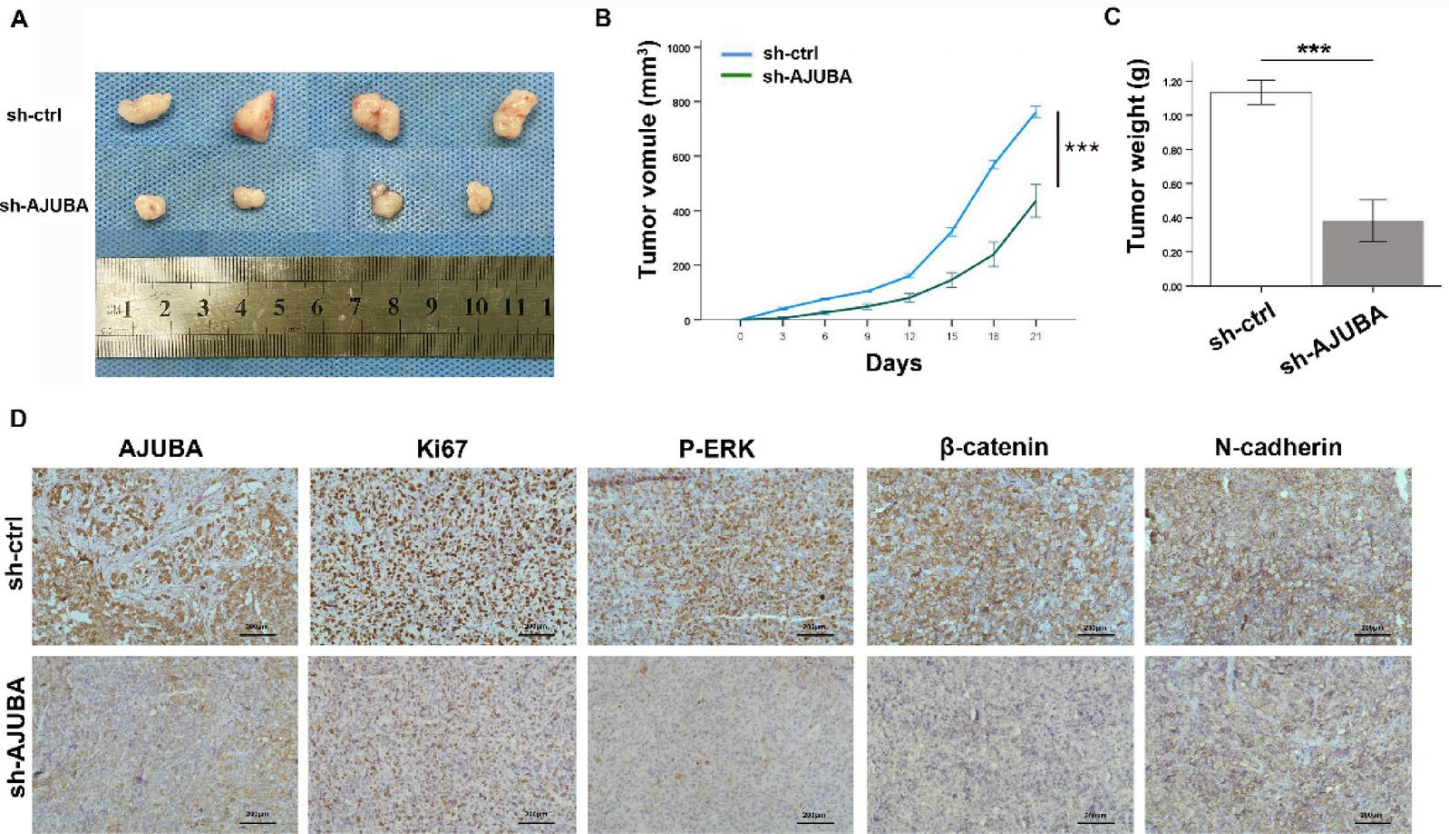
Downregulation of AJUBA inhibits the growth of NSCLC cells in vivo. (**A**) Image of tumors in nude mouse tissues from the two groups. (**B**) Tumor volume in the shctrl and shAJUBA groups. Two-tailed Student’s t-test was used to analyze the differences between the two groups. ^***^P < 0.001. (**C**) The tumor weights in the NSCLC mouse model were measured in the shctrl and shAJUBA groups. Two-tailed Student’s t-test was used to analyze the differences between the two groups. ^***^P < 0.001. (**D**) The expression levels of AJUBA, Ki67, p-ERK, β-catenin and N-cadherin were investigated in control and AJUBA-depleted groups by IHC (200X).

The effect of AJUBA deficiency on NSCLC growth was measured in a mouse model. Mice treated with shAJUBA showed lower tumor growth volumes compared with control mice (Fig. 6A and B). These mice exhibited significantly lower tumor weights than the control group (Fig. 6C). Furthermore, the expression of p-ERK, β-catenin, Ncadherin and Vimentin decreased in the shAJUBA group (Fig. 6D). Taken together, these data indicate that AJUBA promoted tumor growth via regulation of the ERK and Wnt/β-catenin pathway in NSCLC.

## Discussion

Although the diagnostic technology and treatment methods for NSCLC have been continuously improving, its five-year survival rate remains very low^19^. It is necessary to explore the core mechanisms of tumor progression to discover novel biomarkers. Although involvement of AJUBA in several signaling cascades has been reported^20^, and the function of AJUBA investigated in a series of human cancers^16,21–24^, there have been no reports to the best of our knowledge on the in vivo role of AJUBA in lung cancer and its impact on prognosis. In this study, we demonstrated that AJUBA was markedly increased in NSCLC while deletion of AJUBA caused a reduction in the proliferation and motility of NSCLC cells. Furthermore, knockdown of AJUBA regulates growth in both cell lines and mouse models by repressive activity on the Wnt/β-catenin pathway. Our study thus provides new evidence showing AJUBA as a tumor biomarker in NSCLC.

Previous research has found that AJUBA expression is related to poor prognosis in HCC, CRC, breast cancer and esophageal squamous cell carcinoma^6,9,11,12^, but the impact of AJUBA on the survival rate in lung cancer has not been reported. In this study, AJUBA overexpression was observed in 127/188 NSCLC specimens and was associated with progression of existing clinicopathological factors. Survival analysis results showed that AJUBA expression was significantly related to reduced survival. This is consistent with previous results in HCC patients^12^. We also found that siAJUBA treatment induced an anticancer effect on H1299 and A549 cells.

AJUBA is a scaffold or adapter protein containing multiple LIM domains. These domains might identify a shared structural characteristic of proteins, given that there isn’t a universal LIM domain recognition sequence among proteins^3^. Previous studies have shown that AJUBA interacts with various proteins such as Snail^25^, sp1^26^and p62^27^to play multiple roles. β-catenin is one of these proteins, which initially aroused our interest. The Wnt/β-catenin pathway plays a significant role in lung cancer, and currently, there is no research on its regulatory relationship with AJUBA in lung cancer. The Wnt/β-catenin pathway is involved in cellular processes, tumorigenesis and EMT. In HCC, AJUBA is necessary for activation of the EMT pathway and HCC progression by increasing the levels of mesenchymal features^12^. Our present study demonstrated that AJUBA was positively correlated with β-catenin in adenocarcinoma (*p* = 0.012) and squamous cell carcinoma (*p* = 0.014) tissues. Down-regulation of AJUBA led to a decrease in a series of key molecules in EMT as well as downstream genes in the Wnt pathway. Thus, the impact of AJUBA on tumor prognosis may involve regulating the Wnt/β-catenin pathway.

Inappropriate activation of ERK signaling is implicated in lung cancer, and the ERK pathway is necessary to induce EMT progression^28^, while β-catenin is upregulated by p-ERK and contributes to lung cancer metastasis^29^. According to the literature, the earliest functional studies demonstrated that AJUBA binds to Grb2, resulting in activation of the ERK pathway in a RAS-dependent manner^4^. These findings prompted us to focus on the impact of p-ERK levels on the function of AJUBA in lung cancer. In our study, AJUBA overexpression increased malignant phenotypes of NSCLC cell lines, and EMT-related proteins were also increased in the AJUBA overexpression group. Targeting ERK with an inhibitor effectively reduced AJUBA-induced tumor progression. Moreover, ERK dephosphorylation induces downregulation of β-catenin, N-cadherin and Vimentin. The NSCLC mouse model we constructed showed that shAJUBA inhibited the xenograft growth in models using NSCLC cells, and AJUBA ablation suppressed the expression of Ki67, p-ERK, β-catenin, N-cadherin and Vimentin simultaneously in tumor tissues. Thus, AJUBA promotes proliferation, motility and EMT by targeting the ERK and Wnt/β-catenin pathways.

It is worth mentioning that, unlike AJUBA, the other two members of the AJUBA family (Wilms tumor 1 interacting protein and LIMD1) seem to play different roles in lung cancer^30,31^. This may be attributed to their distinct impacts on the same signaling pathways in lung cancer. In NSCLC, high expression of LIMD1 can inhibit the over-activation and nuclear localization of yes-associated protein (YAP), thereby suppressing the progression of lung cancer^32^. In addition to the WNT pathway, previous literature documented that AJUBA could also regulate the Hippo pathway^3^, which is required in many processes such as cell proliferation, EMT and carcinoma formation^20^. In CRC, the Hippo/YAP pathway was activated significantly after AJUBA knockdown and attenuated the growth of CRC cells^23^. High AJUBA levels enhanced cervical cancer cell drug resistance to cisplatin by upregulating YAP and the transcriptional coactivator TAZ. Overexpression of AJUBA also regulated glucose uptake and mitochondrial potential through Hippo/YAP in human gastric cancer^10^. The above conclusion seems to imply that AJUBA has different regulatory effects on YAP in different types of tumors. The relationship between AJUBA and Hippo/YAP in lung cancer has not been reported until now. Because the Hippo and Wnt pathways both affect tumorigenesis, it will be essential to study the cross-talk between AJUBA, WNT and Hippo in NSCLC in future experiments.

AJUBA has functioned as an oncogene in most studies, but also has been shown to have tumor-suppressing effects in certain cancers. For example, AJUBA functions as a haploinsufficient tumor suppressor by promoting NOTCH signaling in head and neck squamous cell carcinomas^33^. AJUBA’s effects are context-dependent and tissue-specific in tumorigenesis, which will require further research.

This study does have some limitations. When we conducted the AJUBA upregulation experiment with the addition of an ERK inhibitor, we found that the experimental dose of the ERK inhibitor did not completely offset the protein upregulation and enhanced tumor malignancy caused by AJUBA upregulation. Since the purpose of this experiment was to verify that upregulation of AJUBA could promote p-ERK expression, we did not use a high concentration of the ERK inhibitor. Meanwhile, the experimental results suggested that AJUBA may also regulate tumor malignancy through ERK-independent pathways, which needs to be confirmed in future studies. The in vivo experiments in this study did confirm that AJUBA inhibited tumor growth and we observed a reduction in p-ERK in vivo. To further verify whether AJUBA inhibits tumor growth in vivo through the suppression of the ERK pathway, the next step should include mouse experiments with the injection of ERK inhibitors. Furthermore, in vivo experiments need to be conducted to verify whether AJUBA can be used as a gene target for the treatment of NSCLC. This should involve conducting dose-response experiments with AJUBA inhibitors, exploring AJUBA’s role in resistance to existing chemical therapies, and validating findings across a broader range of NSCLC subtypes.

In conclusion, our results suggest that AJUBA is commonly and significantly up-regulated in NSCLC, which suggests an important role in the acquisition of a poor prognostic phenotype. AJUBA promotes the growth of lung cancer cells both in vitro and in vivo. AJUBA also accelerates EMT in NSCLC by inducing the ERK and Wnt/β-catenin pathways. Our findings thus indicate that AJUBA may be a key prognostic marker in NSCLC. This new understanding of the underlying mechanisms involving the AJUBA and Wnt/β-catenin pathways will provide a potential new target for the treatment of NSCLC.

## Materials and methods

### Immunohistochemistry

A collection of 188 paired tumor and paracancerous paraffin tissues without any preoperative treatment were used for IHC staining and were purchased from the Shanghai Outdo Biotech Company. The study was performed in strict compliance with the Declaration of Helsinki and was approved by the Ethics Committee of the Taizhou Hospital of Zhejiang province (SHYJS-CO-1904014,1910013). Sections were subjected to dewaxing with xylene and gradient alcohol dehydration followed by antigen retrieval using heat-mediated antigen retrieval in citrate buffer. The sections were blocked with normal goat serum (Maixin) for 10 min at room temperature. Subsequently, the sections were incubated with primary anti-AJUBA rabbit polyclonal antibody (HPA006171; 1:100, Sigma), anti-β-catenin (ab32572;1:400, Abcam), anti-Ncadherin (ab76011; 1:400, Abcam), anti-p-ERK (4370; 1:400, Cell Signaling Technology), or anti-Ki67 (RMA-0542; ready-to-use, Maixin) at 4 °C. The next morning, the sections were incubated with the Elivision super Kit from Maixin (KIT-9921). DAB-2031 (Maixin) was added for 2 min to visualize the proteins. The intensity of staining of AJUBA was graded as follows: 1 (no staining), 2 (light yellow) and 3 (deep yellow). The percentage of staining was assigned as 1 (< 10%), 2 (10–50%) and 3 (51–100%). Scores for intensity and percentage of staining were assigned to each sample and then multiplied together. Resulting values ≥ 4 were considered to indicate high expression. The intensity of staining scores for β-catenin were assigned based on previous studies^34^. The criteria for differentiation were as follows: For adenocarcinoma, if the proportion of high-grade components (solid, micropapillary, cribriform and complex glandular structures) was less than or equal to 20%, it was considered well-differentiated. If the proportion was greater than 20%, it was considered poorly differentiated. For squamous cell carcinoma, the presence of keratin pearls and intercellular bridges in cells with incomplete keratinization indicated good differentiation, while the absence of those features indicated poor differentiation. Two independent experienced investigators were assigned to examine all tumor slides randomly.

### Cell culture

In this study, all NSCLC cell lines and the HBE cell line were maintained in DMEM/F12 (1:1) medium (Gibco), with 10% FBS (Biological Industries) at 37 °C under 5% CO_2_. For transfection, negative control siRNA and siAJUBA#1 (SR313776) were purchased from Origene. siAJUBA#2 5’-GGACCGGGA.

UUAUCACUUUTT-3 was synthesized by Gene Pharma (Shanghai). For AJUBA overexpression, NSCLC cells were transfected with Plasmid Control(PC) and pCMV6-AJUBA plasmid (RC215384, Origene). Lipofectamine 3000 (Invitrogen) was used as transfection reagent. Transfected cells were used for further experiments after 48 h.

### Real-time PCR

TRIzol was used to extract total RNA from the samples. Real-time PCR was conducted using Supermix from Bio-Rad in a 20-ul reaction system, with triplicate samples analyzed on the Light Cycler^®^480 II. GAPDH was used as an internal reference, and relative quantification of all samples was performed using the 2^−ΔΔCT^ method. The sequences of the primers are listed in Table S1.

### Cell counting Kit-8 (CCK-8) assays

After 24 h of transfection with siAJUBA or pCMV6-AJUBA plasmids, NSCLC cells were seeded in 96-well plates (5 × 10^3^ cells per well). CCK-8 reagent (10 µl; Beyotime) was added 4 h later and the cells were incubated for an additional 2 h at 37 °C. OD values were read at a wavelength of 450 nm using a full-wavelength microplate reader (Thermo Fisher Scientific). Growth curves were plotted using data from all observation time points, and statistical analysis was performed using data from the end point of observations.

### Colony formation assay

H1299 or A549 cells (1 × 10^3^ cells/well) were transfected with siAJUBA or pCMV6-AJUBA plasmids in 6-well plates. Crystal violet (Solarbio, Beijing, China) was used to stain the cell colonies after 14 days.

### Matrigel invasion assay

Migration experiments were conducted using 24-well plates, with matrix gel (BD) diluted 1:6 in serum-free culture medium. Cells were incubated at 37 °C 16 h, then cells which had not invaded the lower layer were removed using a cotton tip. The cells that had passed through the membrane were fixed with formalin for 15 min and then stained with crystal violet for 15 min. Photos were taken and statistical analysis was performed on 10 randomly selected high-power microscopic fields for each sample.

### Cell migration assay

H1299 and A549 cells were seeded into 6-well plates 48 h after transfection. Once the cells were confluent, a sterile 20-µL pipette tip was used to scratch the cell surface. The cells were observed and photographed by a digital camera at the same position at 0 h and 24 h later. The scratch width was measured and quantitatively analyzed with Image-Pro Plus 6.0 software.

### Protein Immunoblotting experiment

Total protein was isolated from cells by M-PER™ (78503; Thermo Fisher Scientific) with Halt Protease and Phosphorylase Inhibitor Cocktail (78441; Thermo Fisher Scientific), then quantified using Pierce™ BCA assays (23225; Thermo Fisher Scientific). Sample buffer was added to the protein mixture to achieve a total protein concentration of 60 ug per 20 ul, and the cells were incubated at 100 ˚C for 5 min to denature the protein. After 10% SDS-PAGE, we use semi-dry transfer to move the proteins onto a PVDF membrane (Millipore) with a pore size of 0.45 mm. Next, non-specific binding was blocked with 5% milk and the membrane was incubated at 4℃ overnight with one of the following primary antibodies: AJUBA (HPA0061710; 1:1000, Sigma); Cyclin D1 (2978), Matrix Metalloproteinase-9 (MMP-9; 13667), ERK (4695) or p-ERK (4370, all 1:1000 from Cell Signaling Technology); β-catenin (ab32572), N-cadherin (ab76011) and Vimentin (ab92547), all 1:1000 from Abcam); and GAPDH (1:3000, Affinity Biosciences). The next day, after thorough washing, the membranes were incubated with the appropriate secondary antibodies at 37℃ for 90 min. Chemiluminescence reagent (Bio-Rad) was used to visualize the protein bands with the ECL detection system (Bio-Rad). Image Lab™ Software (Bio-Rad) was used to quantify the relative density of the bands. Images of full-length, original, unprocessed blot densitometric data and calculations of significance are provided in the supplementary materials.

### Xenograft tumor assay

Female nude mice (body weight: 18–20 g) were obtained from Liaoning Changsheng Biotechnology and were meticulously cared for in accordance with the Laboratory Animal Care protocols established by the First Hospital of China Medical University. These protocols are aligned with the recommendations from the US National Institutes of Health and adhere to the ARRIVE guidelines, which can be accessed at the following link (https://arriveguidelines.org) for further details. The mice were allowed to adapt to a specific pathogen-free environment with a temperature range of 23 to 24˚C, relative humidity levels between 30 and 50%, and a 12-h alternating light/dark cycle. During the entire experimental process, food and water were freely available. H1299 cells were transfected with lentiviral supernatant containing shRNA targeting AJUBA (TL303846, Origene) or shControl (TR30021, Origene). Stable cell lines were positively selected using 34 ug/ml Chloramphenicol. The mice were divided into the shAJUBA group and the shCtrl group. Each experimental group was implanted with 5 × 10^6^ stably transfected H1299 cells. Measurement of tumor nodules was performed once per week. The tumor volume was calculated using the following formula: Tumor Volume = Longest Diameter × (Shortest Diameter/2)/2. All mice were euthanatized 3 weeks later, at which time the tumor tissues were dissected and weighed.

### Statistical analysis

All statistical analyses were conducted using SPSS 27.0 software (IBM). The specific statistical methods used in each experiment are explained in the text and figure legends. Data are presented as means plus or minus standard deviation (SD). The levels of statistical significance are denoted by p-values.

## Electronic supplementary material

Below is the link to the electronic supplementary material.

Supplementary Material 1

Supplementary Material 2

Supplementary Material 3

Supplementary Material 4

## Data Availability

The datasets used and/or analyzed during this study are available from the corresponding author upon reasonable request.

## Abbreviations

(si)RNA: Silencing RNA
TNM: Tumor Node Metastasis stage
ERK: Extracellular Signal-Regulated kinases
EMT: Epithelial-Mesenchymal Transition
WTIP: Wilms Tumor 1Interacting Protein
LIMD1: LIM Domain-Containing Protein 1
CRC: Colorectal Cancer
ESCC: Esophageal Squamous Cell Carcinoma
HCC: Hepatocellular Carcinoma
IHC: Immunohistochemistry
HBE: Human Bronchial Epithelial
FBS: Fetal Calf Serum
DMEM: Dulbecco’s Modified Eagle’s Medium
NC: Negative Control
PC: Plasmid Ccontrol
GAPDH: Glyceraldehyde-3-Phosphate Dehydrogenase
CCK-8: Cell Counting Kit-8
MMP-9: Matrix Metalloproteinase-9
