# Supplementary material for: AJUBA promotes the proliferation, invasion and migration of NSCLC cells by activating the ERK/β-catenin pathway
Source: Sci Rep. 2025 Apr 16;15:13123. doi: 10.1038/s41598-025-98156-z (PMC12003803; doi:10.1038/s41598-025-98156-z)
Supplement: Supplementary file 1 — Supplementary Material 1 [file 41598_2025_98156_MOESM1_ESM.docx]

| Name | Primer sequences |
| --- | --- |
| AJUBA forward | 5′-GATGCGGGAGCCAGAGG-3′ |
| AJUBA reverse | 5′-CACAAGAGCAGCAAACAAAGC-3′ |
| GADPH forward | 5′-ACAACTTTGGTATCGTGGAAGG-3′ |
| GADPH reverse | 5′-GCCATCACGCCACAGTTTC-3′ |

Supplement Table1 Primer information
