# Supplementary material for: AJUBA promotes the proliferation, invasion and migration of NSCLC cells by activating the ERK/β-catenin pathway
Source: Sci Rep. 2025 Apr 16;15:13123. doi: 10.1038/s41598-025-98156-z (PMC12003803; doi:10.1038/s41598-025-98156-z)
Supplement: Supplementary file 2 — Supplementary Material 2 [file 41598_2025_98156_MOESM2_ESM.pdf]

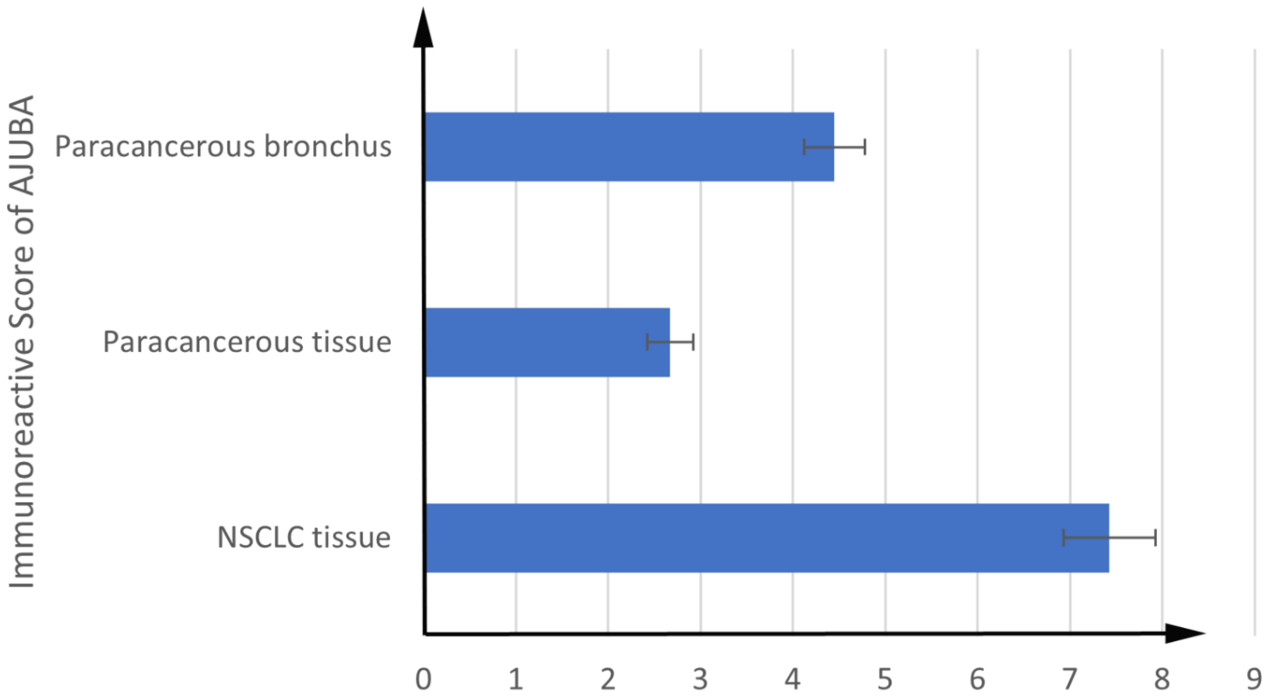

Supplementary Figure 1. The protein level of AJUBA in NSCLC tissues was detected by immunohistochemical staining assay.

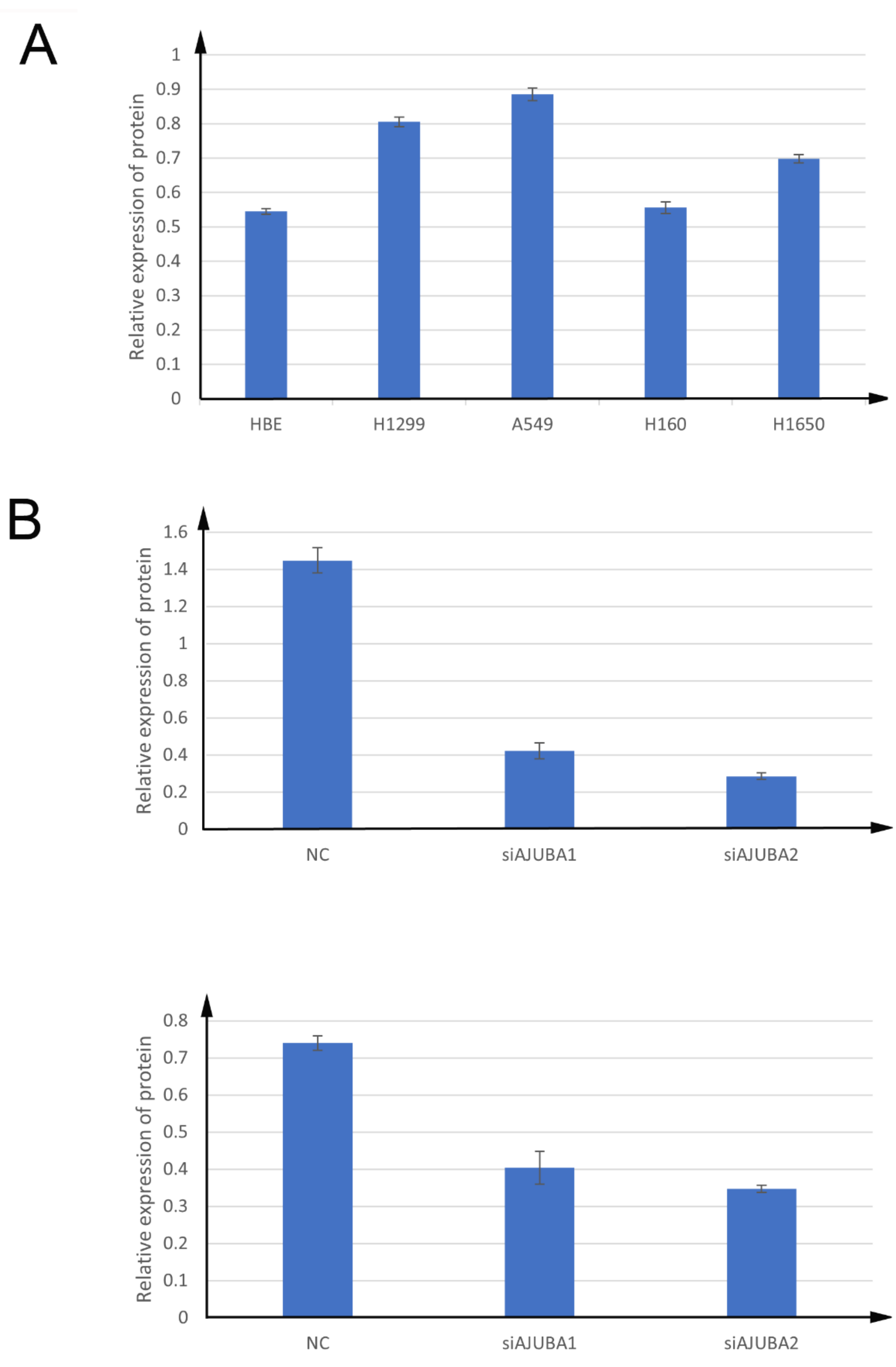

Supplementary Figure 2. Protein expression of AJUBA in each group of cells.

(A) Protein expression levels of AJUBA in normal lung epithelial cells (HBE) and NSCLC cell lines. **(B)** The protein levels of AJUBA in AJUBA-knockdown H1299 (Up) and A549 (down) cells.

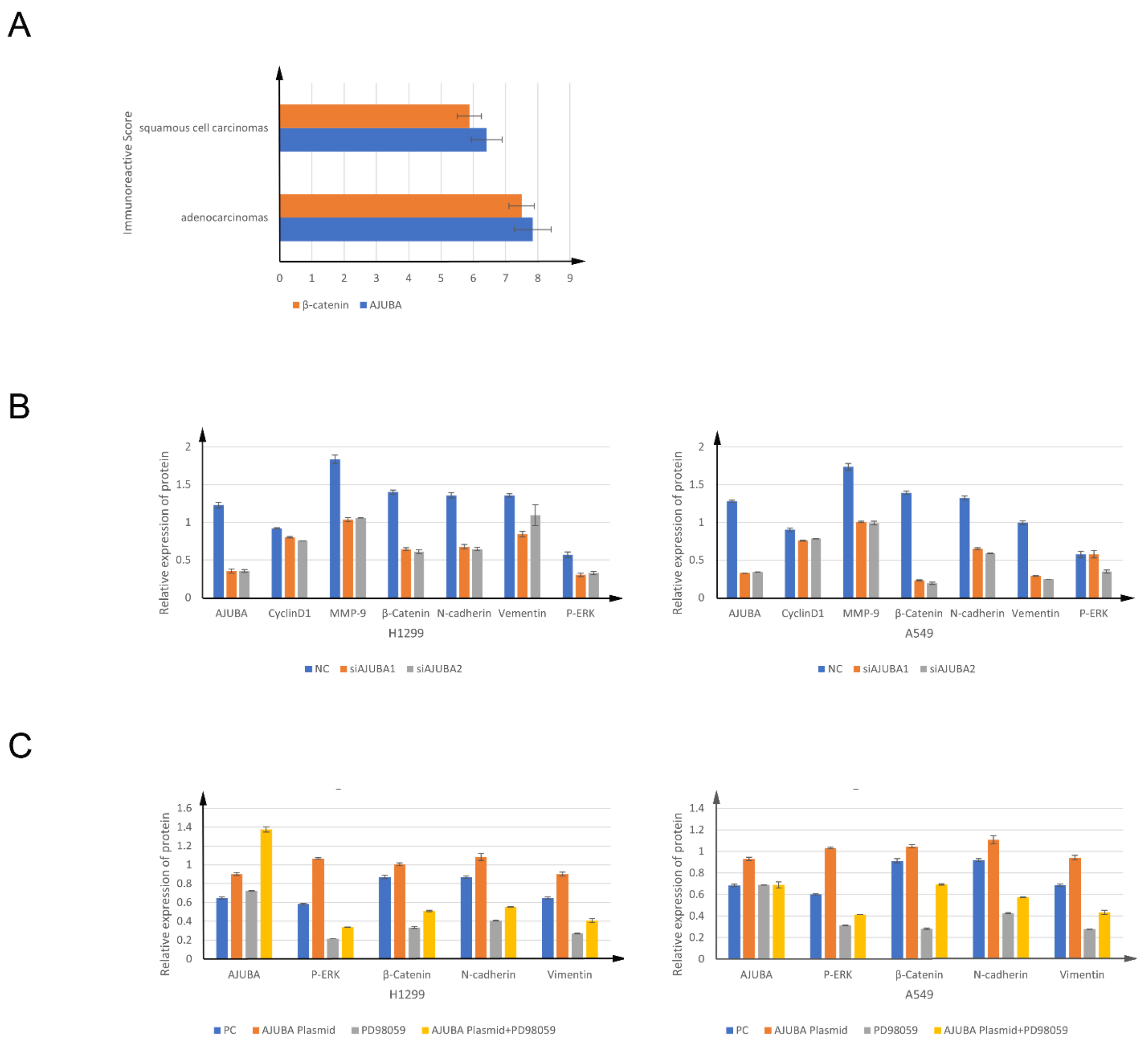

Supplementary Figure 3. Correlation between AJUBA and EMT-related proteins expression. (A) Correlation of the protein level of AJUBA and β-catenin in NSCLC tumor tissues by immunohistochemical staining assay. **(B)** Western blot analysis of the expression of β-catenin signaling in H1299 (left) and A549 (right) cells after transfection with or without siAJUBA. **(C)** Western blot analysis of the expression of β-catenin signaling in H1299 (left) and A549 (right) cells with AJUBA overexpression and/or PD98059 treatment.
