# Supplementary material for: AJUBA promotes the proliferation, invasion and migration of NSCLC cells by activating the ERK/β-catenin pathway
Source: Sci Rep. 2025 Apr 16;15:13123. doi: 10.1038/s41598-025-98156-z (PMC12003803; doi:10.1038/s41598-025-98156-z)
Supplement: Supplementary file 3 — Supplementary Material 3 [file 41598_2025_98156_MOESM3_ESM.pdf]

Figure2 A

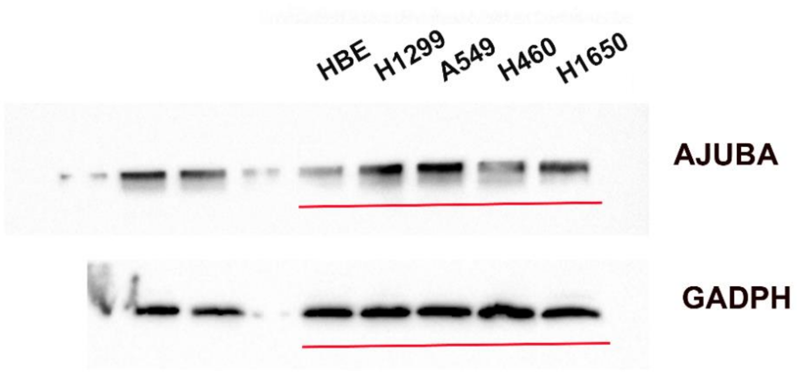

Figure 2 C

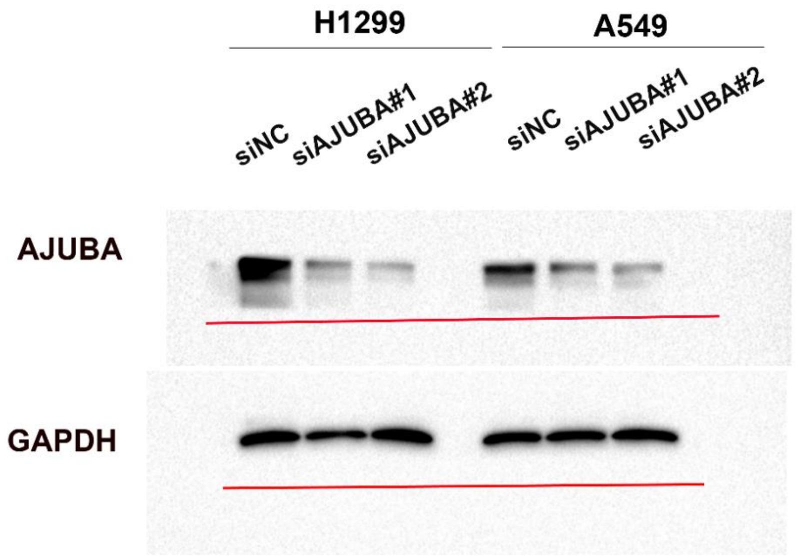

Figure 4 B -AJUBA

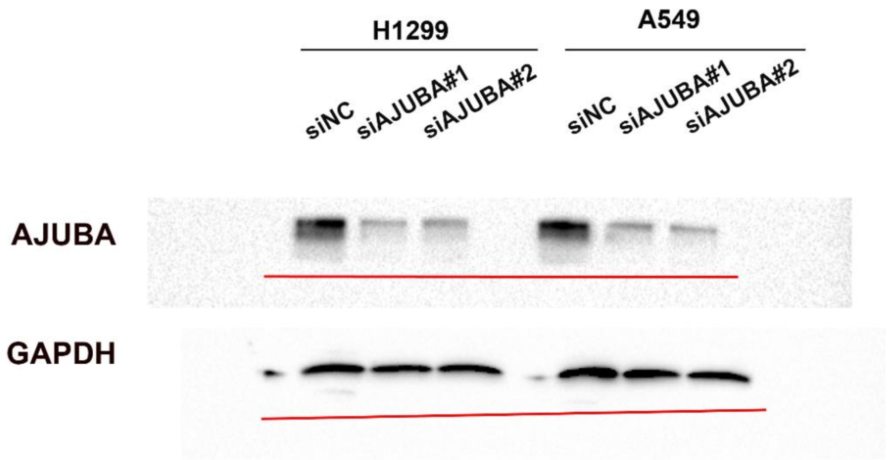

Figure 2 and 4 WESTERN BLOT ORIGINAL IMAGES

Figure 4B-Cyclin D1

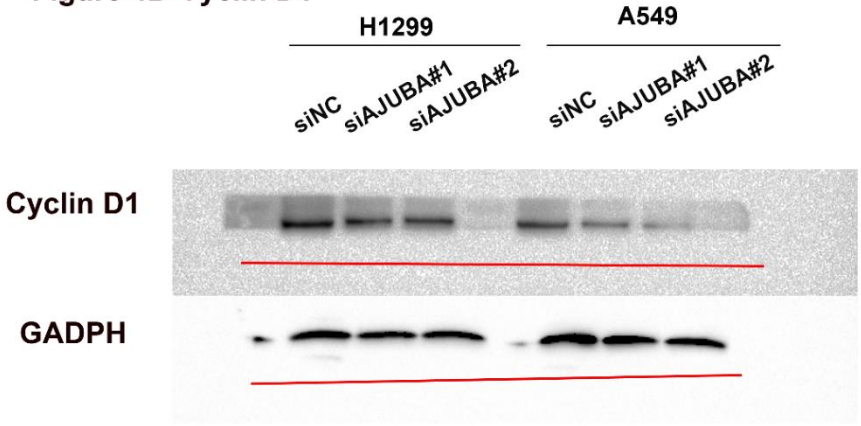

Figure 4 B - MMP-9

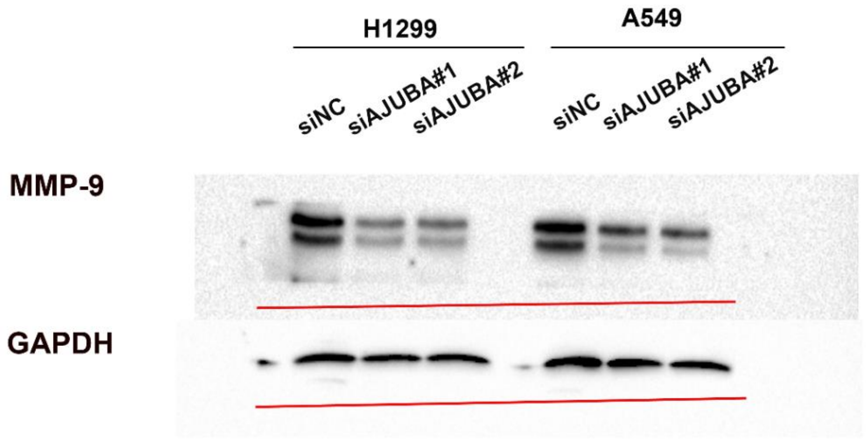

Figure 4 B - Vimentin

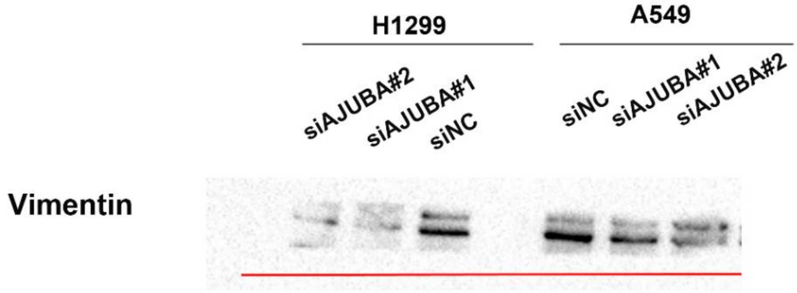

Figure 4 WESTERN BLOT ORIGINAL IMAGES

Figure 4 B - β-Catenin

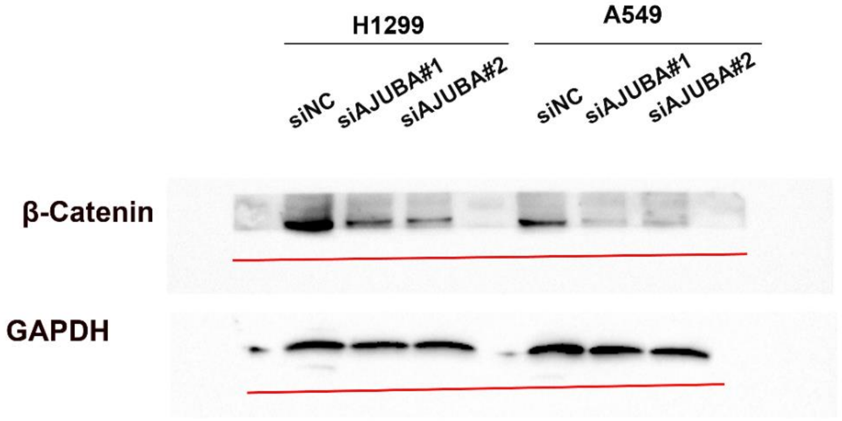

Figure 4 B P-ERK

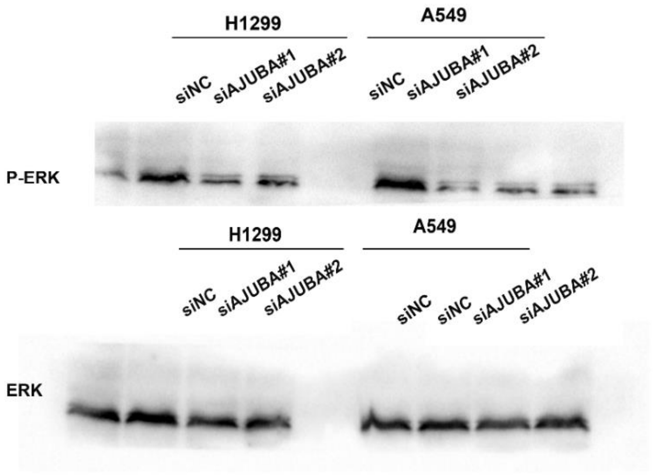

Figure 4 WESTERN BLOT ORIGINAL IMAGES

Figure 4C-AJUBA

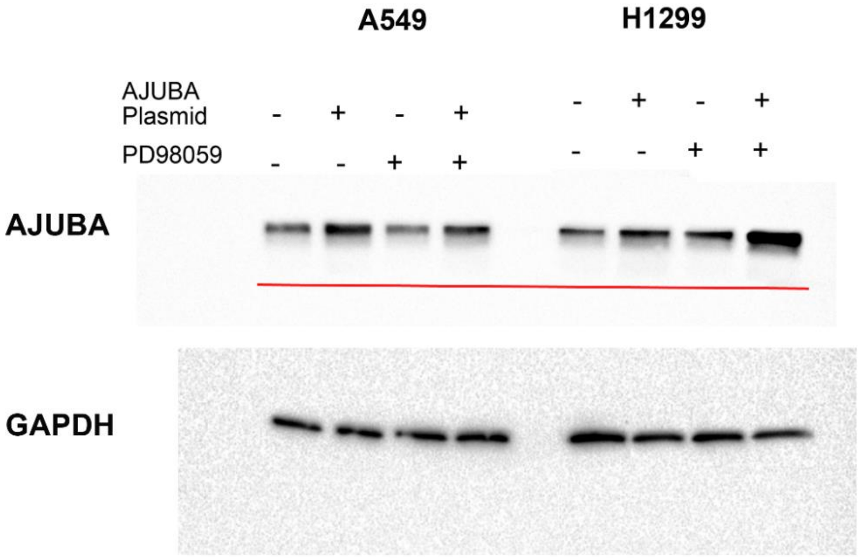

Figure 4C-.P-ERK

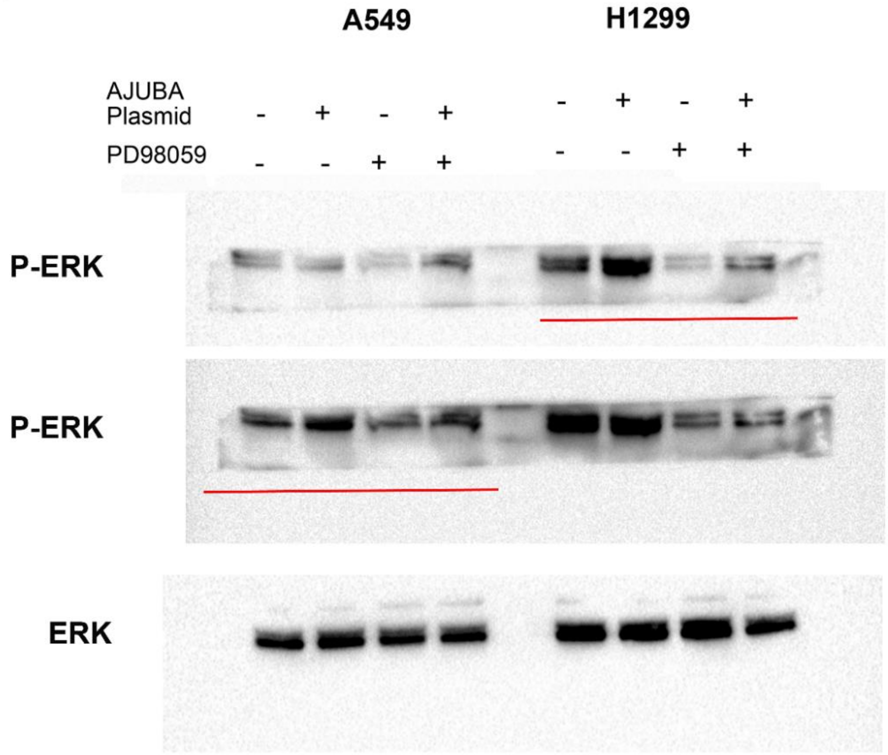

Figure 4 WESTERN BLOT ORIGINAL IMAGES

Figure 4C- N-cadherin

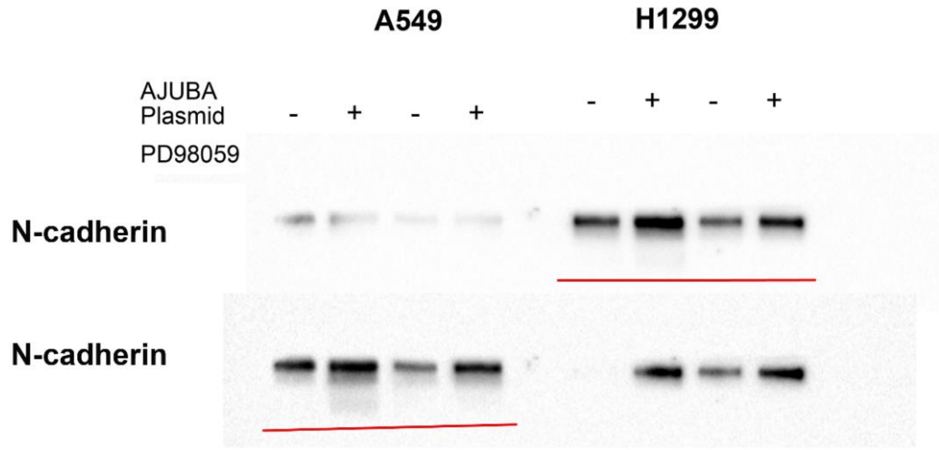

Figure 4C- Vimentin

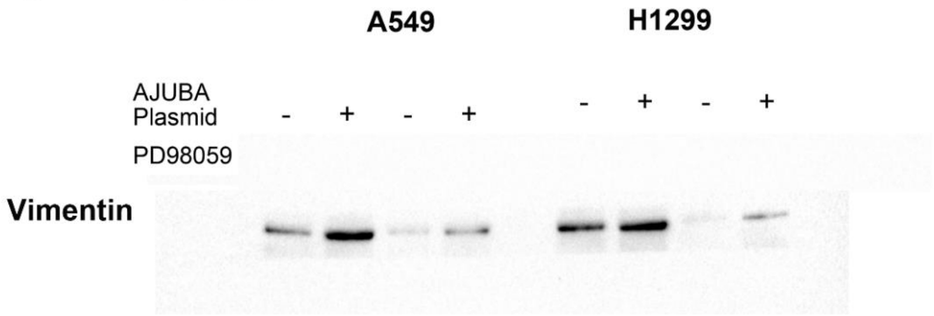

Figure 4C- β-Catenin

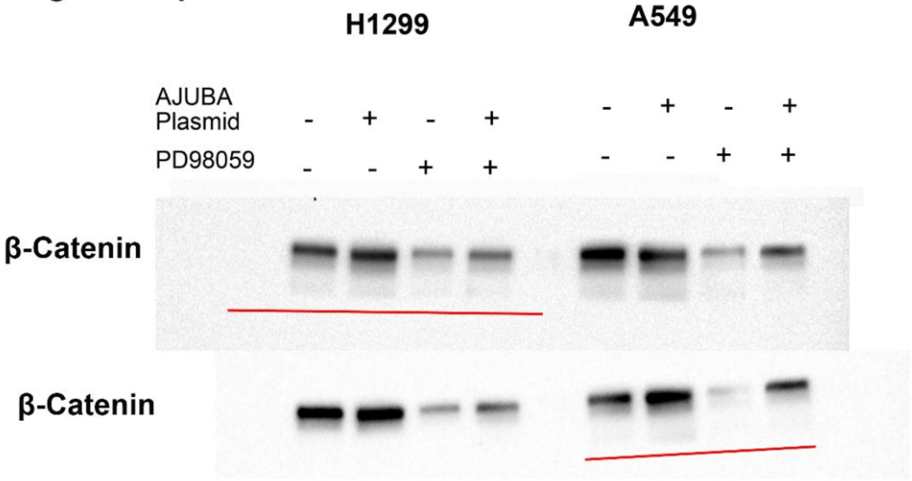

Figure 4 WESTERN BLOT ORIGINAL IMAGES
