# Supplementary material for: AJUBA promotes the proliferation, invasion and migration of NSCLC cells by activating the ERK/β-catenin pathway
Source: Sci Rep. 2025 Apr 16;15:13123. doi: 10.1038/s41598-025-98156-z (PMC12003803; doi:10.1038/s41598-025-98156-z)
Supplement: Supplementary file 4 — Supplementary Material 4 [file 41598_2025_98156_MOESM4_ESM.pdf]

Figure 2 and 4 WESTERN BLOT ORIGINAL IMAGES

Fig2A-AJUBA

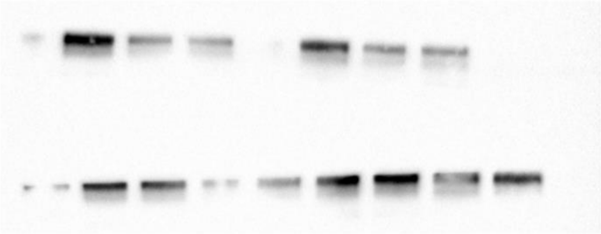

Fig2A-GAPDH

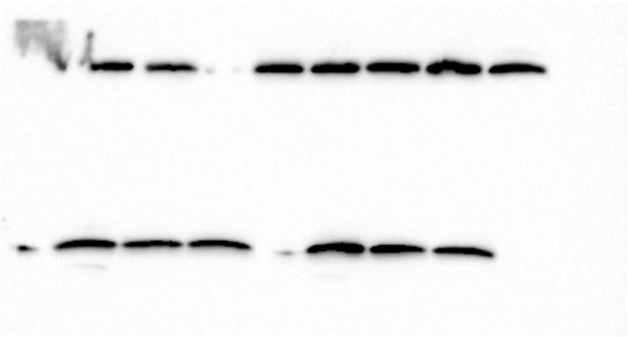

Fig2C-AJUBA

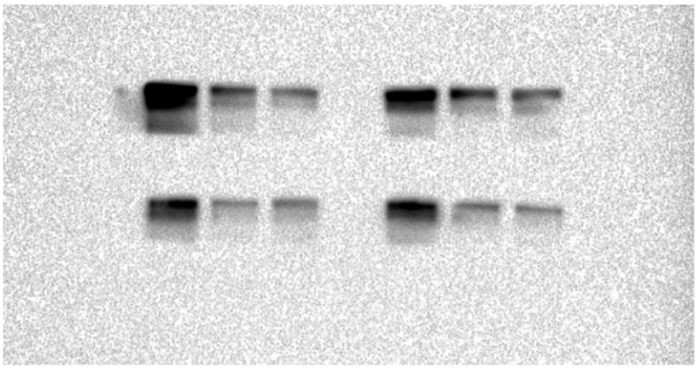

Fig2C-GADPH

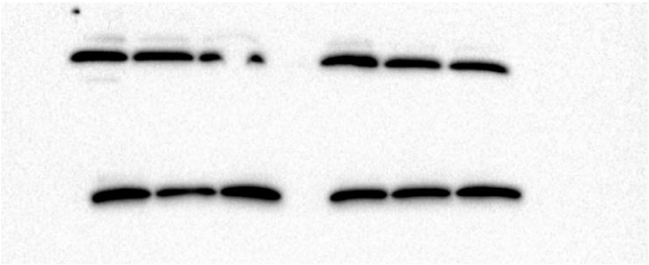

Figure 2 WESTERN BLOT ORIGINAL IMAGES

Fig4B-AJUBA

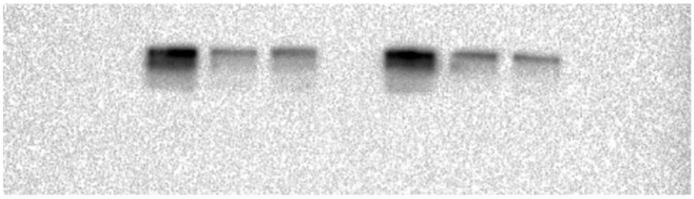

Fig4B-MMP9

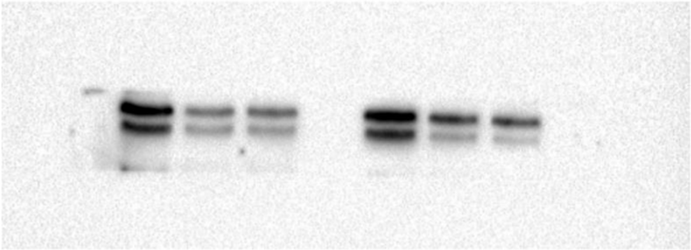

Fig4B-Vimentin

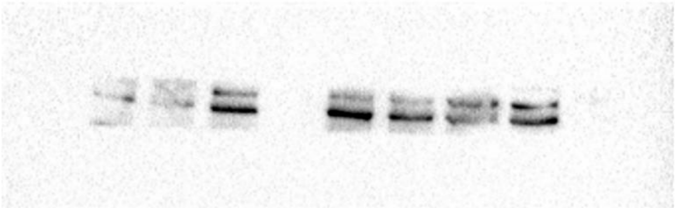

Fig-4B-GAPDH

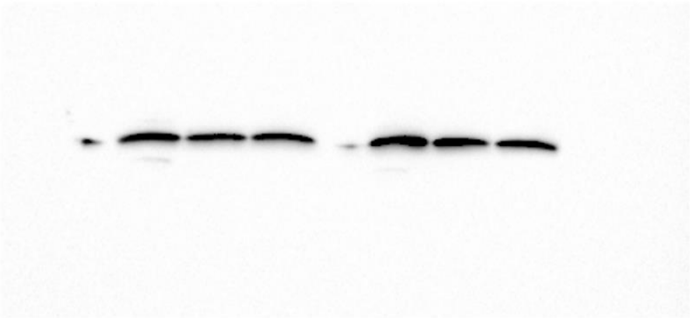

Figure 4 WESTERN BLOT ORIGINAL IMAGES

Fig4B-N-cadherin

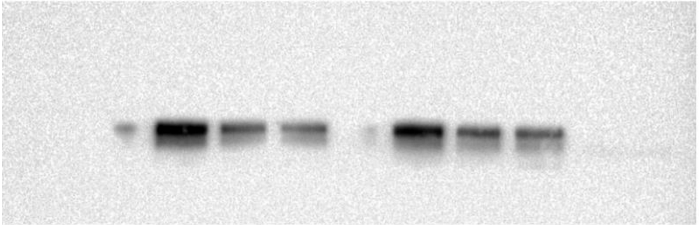

Fig4B-Cyclin D1

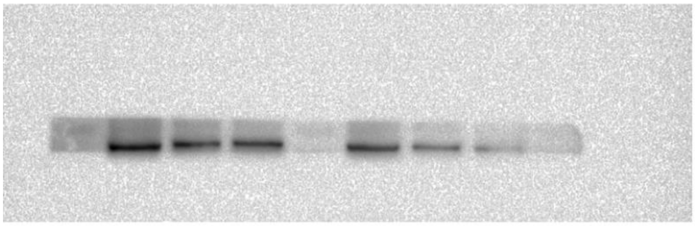

Fig4B-P-ERK

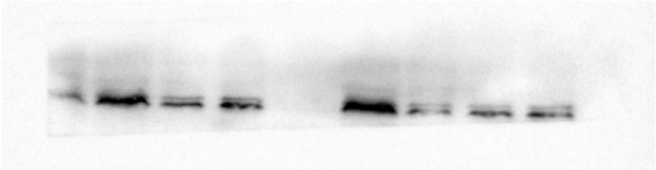

Fig4B-ERK

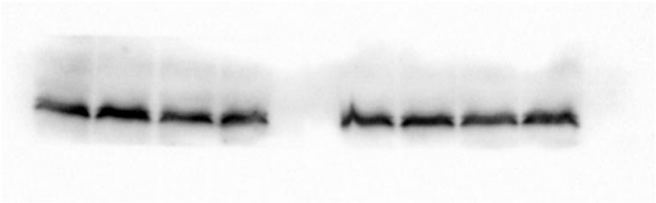

Figure 4 WESTERN BLOT ORIGINAL IMAGES

Fig4C-AJUBA

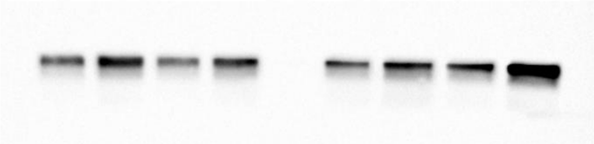

H1299-β-catenin

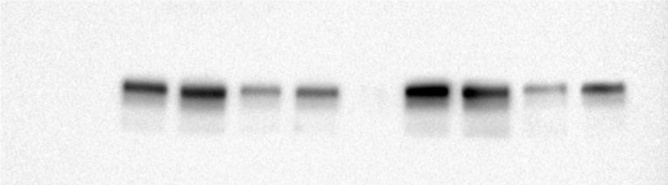

A549-β-catenin

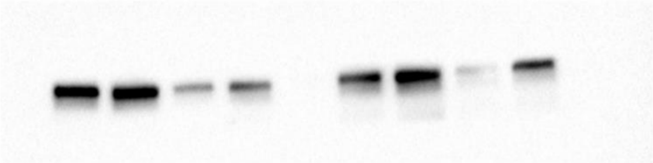

A549- N-cadherin

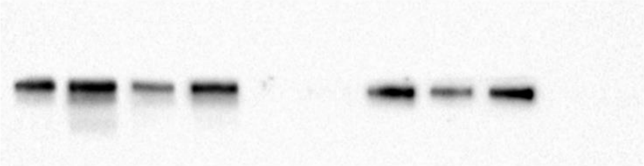

H1299- N-cadherin

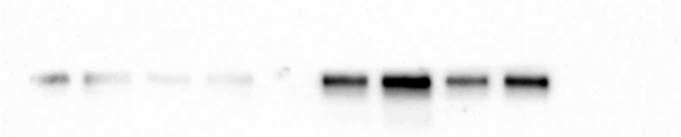

Figure 4 WESTERN BLOT ORIGINAL IMAGES

Fig4C-Vimentin

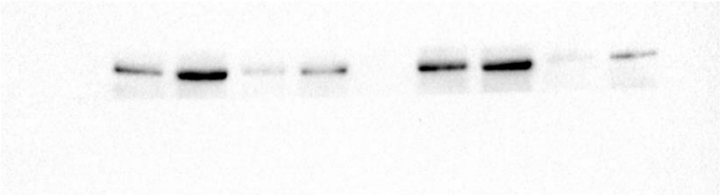

H1299-P-ERK

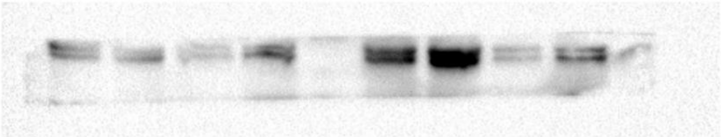

A549-P-ERK

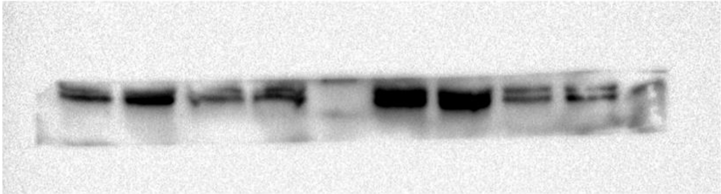

ERK

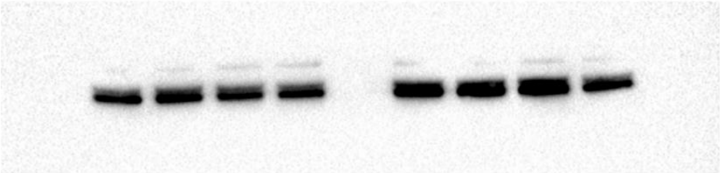

GAPDH

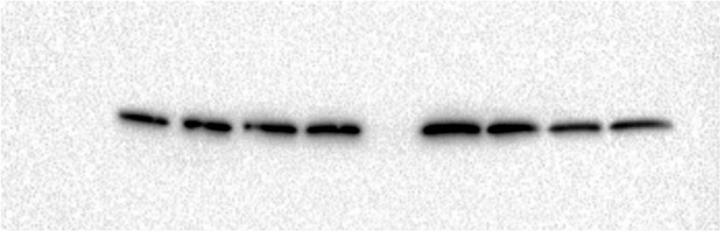

Figure 4 WESTERN BLOT ORIGINAL IMAGES
